# Primary Pulmonary Epithelioid Angiosarcoma With Partial Response to Paclitaxel Treatment: A Case Report

**DOI:** 10.7759/cureus.93687

**Published:** 2025-10-02

**Authors:** Yoshihiro Yamamoto, Hiroki Matsuoka, Saya Tsukida, Yoshimichi Ueda

**Affiliations:** 1 Medicine, Ushitsu General Hospital, Noto, JPN; 2 Respiratory Medicine, Keiju Medical Center, Nanao, JPN; 3 Respiratory Medicine, Kaga Medical Center, Kaga, JPN; 4 Pathology, Keiju Medical Center, Nanao, JPN

**Keywords:** case report, chemotherapy, epithelioid angiosarcoma, paclitaxel, pulmonary angiosarcoma

## Abstract

Pulmonary epithelioid angiosarcoma is a rare, highly aggressive malignancy for which established treatment protocols are lacking. Here, we report the case of a 73-year-old male diagnosed with primary pulmonary epithelioid angiosarcoma with multiple metastases. While paclitaxel is effective for angiosarcoma, its use in pulmonary epithelioid angiosarcoma, to our knowledge, has not been previously reported. Surgery and radiotherapy were not feasible in this patient due to the advanced disease stage. Although disease progression ultimately occurred, weekly paclitaxel monotherapy achieved a clinically meaningful partial response. This case highlights that paclitaxel monotherapy selected based on the patient's overall condition can be a rational therapeutic choice to achieve objective tumor control in frail patients, serving as a valuable benchmark for individualized treatment selection in the absence of a standard treatment.

## Introduction

Pulmonary epithelioid angiosarcoma is an extremely rare and aggressive malignancy with a poor prognosis. Angiosarcoma accounts for only 1-2% of all soft tissue sarcomas, highlighting the rarity of this specific subtype [1]. It generally presents with nonspecific symptoms such as hemoptysis, cough, chest pain, and weight loss, making early diagnosis challenging. Imaging findings are often inconclusive, as the tumor may appear as a solitary mass or as multifocal lesions [2]. A definitive diagnosis necessitates immunohistochemical analysis showing positivity for endothelial cell markers, such as CD31, CD34, and factor VIII-related antigen [3]. Treatment options include surgical resection, chemotherapy, and radiation therapy, although responses to these treatments are often poor, and no standard protocols exist [4]. Paclitaxel, a microtubule inhibitor, is commonly used to treat angiosarcoma; however, to our knowledge, its application in pulmonary epithelioid angiosarcoma has not been previously reported. Here, we report a case of primary epithelioid angiosarcoma that partially responded to paclitaxel treatment.

## Case presentation

A 73-year-old man with hyperuricemia and a gastric ulcer undergoing treatment at a local clinic was referred to our department. The patient had a 53-pack-year smoking history and worked as a carpenter until the age of 68 years. He had no history of asbestos exposure and presented with a one-month history of cough and anorexia, which progressively led to difficulty eating. On his first visit, his Eastern Cooperative Oncology Group Performance Status score was two.

**Figure 1:**
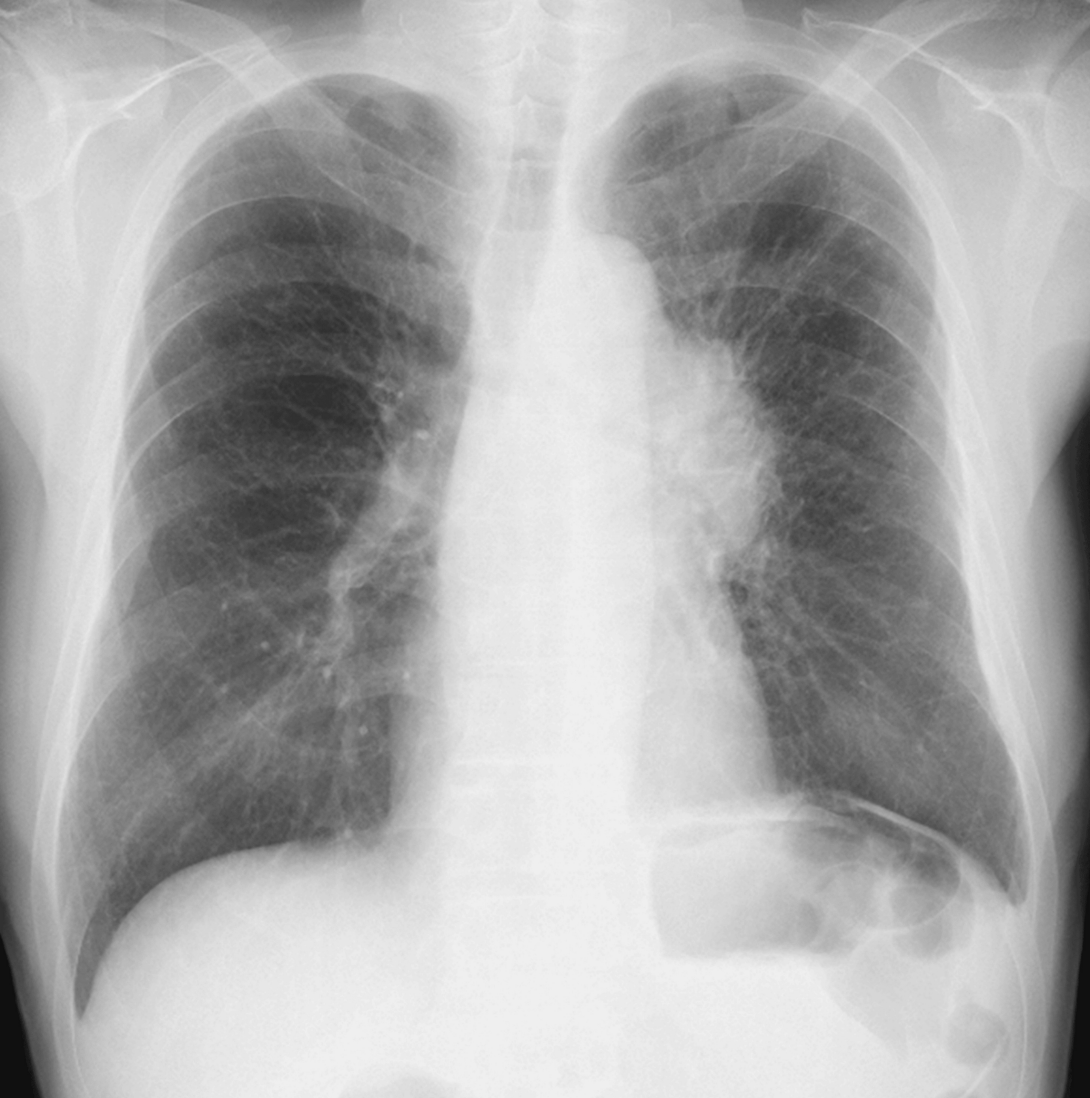
Initial chest radiograph Upright posteroanterior chest radiograph at presentation shows a large left hilar mass.

**Figure 2:**
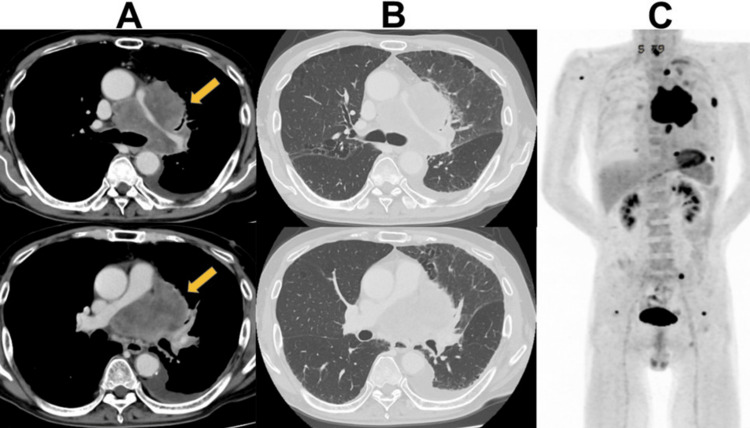
Radiographic findings at initial presentation (A) An axial contrast-enhanced computed tomography image (mediastinal window) demonstrates a large primary tumor in the left hilum with encasement of the pulmonary artery (orange arrow). (B) The corresponding lung window image illustrates the interface between the large tumor and the surrounding lung parenchyma. (C) Positron emission tomography-computed tomography (PET-CT) maximum intensity projection image confirms the extent of the disease, showing multiple sites of high fluorodeoxyglucose (FDG) uptake indicative of widespread metastases.

An initial chest radiograph revealed a large left hilar mass, which prompted further evaluation (Figure 1). A contrast-enhanced computed tomography (CT) scan of the chest revealed a mass measuring 72 mm, extending from the left hilar region to the mediastinum (Figures 2A, 2B). The mass encircled the left pulmonary artery, causing circumferential narrowing, and was in semicircular contact with the descending thoracic aorta. Additionally, bilateral pleural thickening and a small pleural effusion on the left side were noted. Positron emission tomography-CT (PET-CT) revealed metastases in the left lower lung, left pleura, multiple mediastinal lymph nodes, pericardium, bone, and left adrenal glands (Figure 2C), and contrast-enhanced magnetic resonance imaging (MRI) confirmed the absence of brain metastasis. Furthermore, multiple gastric ulcers were observed on upper gastrointestinal endoscopy, requiring the intensification of gastric medication.

**Figure 3:**
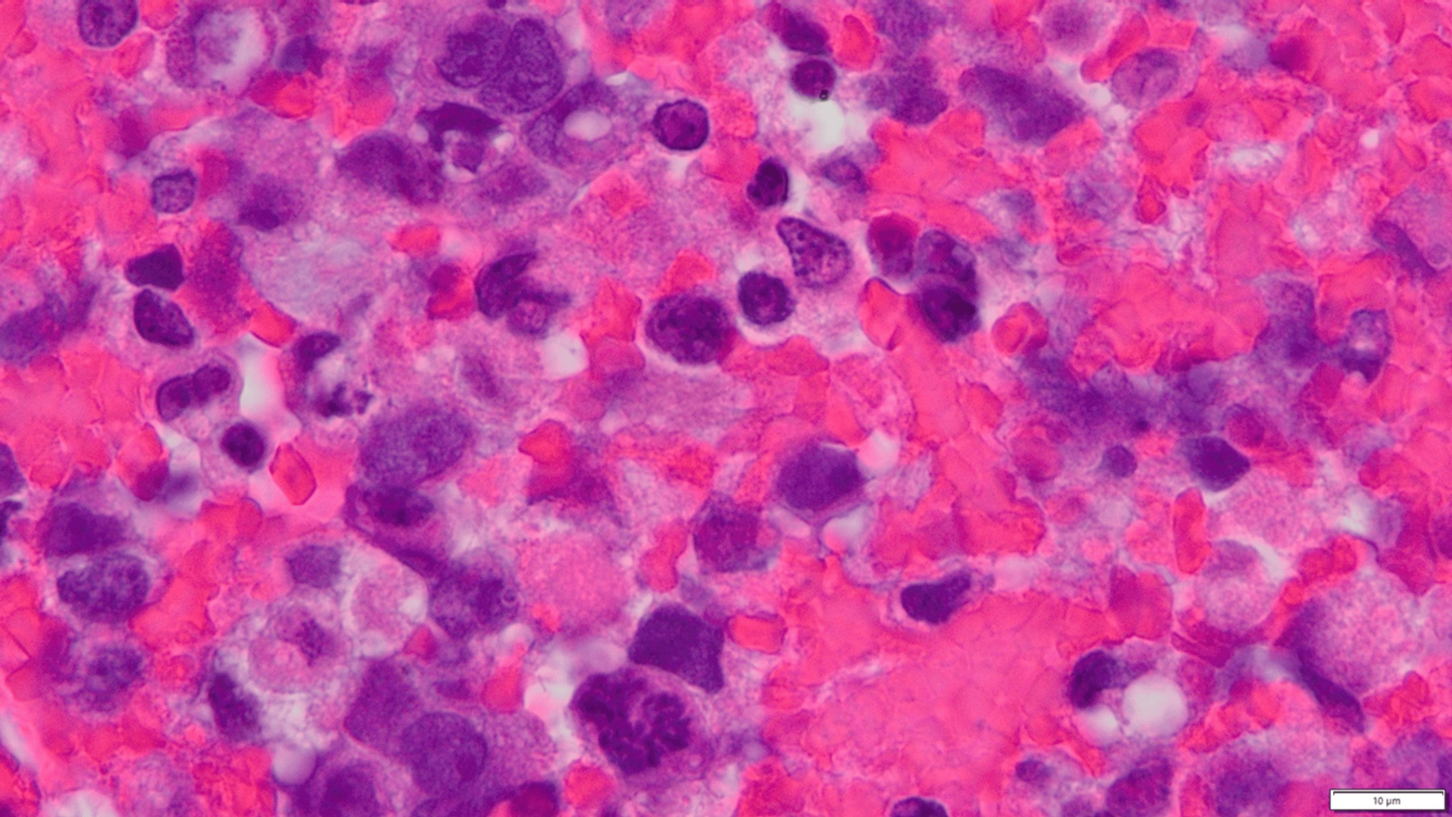
Histopathological examination Hematoxylin and eosin (HE) staining showing an invasive proliferation of large, atypical cells with mild pleomorphism, abundant cytoplasm, and prominent nucleoli. Some cells form irregular, primitive vascular channels containing red blood cells within their cytoplasm.

**Figure 4:**
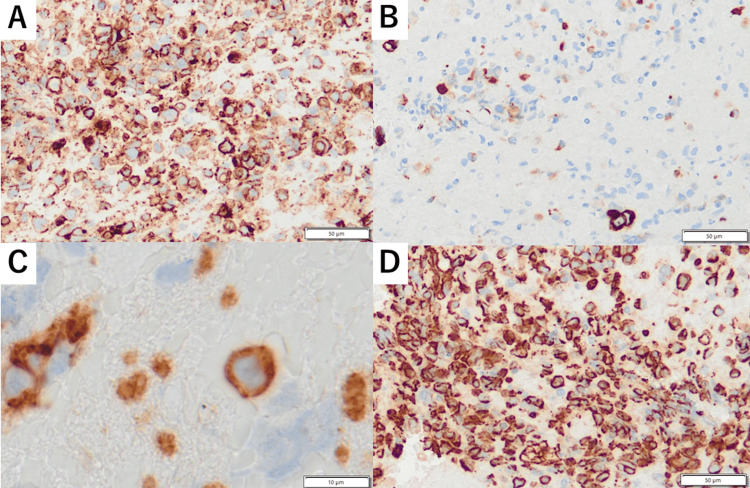
Immunohistochemical examination Tumor cells were positive for (A) vimentin and (B) pan-cytokeratin. The vascular endothelial markers (C) CD31 and (D) CD34 were strongly positive, confirming the endothelial origin of the tumor.

Subsequently, the patient underwent endobronchial ultrasound-guided transbronchial needle aspiration (EBUS-TBNA) of a mediastinal lymph node. Hematoxylin and eosin staining revealed an invasive proliferation of large, atypical cells with mild pleomorphism, abundant cytoplasm, and prominent nucleoli. Some cells formed irregular, primitive vascular channels containing red blood cells within their cytoplasm (Figure 3). Immunohistochemical staining was negative for lung cancer markers, such as thyroid transcription factor-1, Napsin A, and p40. Additional testing revealed that the tumor cells expressed vimentin, pan-cytokeratin, and vascular endothelial markers CD31 and CD34, ruling out alternative diagnoses and leading to a diagnosis of epithelioid angiosarcoma (Figure 4).

**Figure 5:**
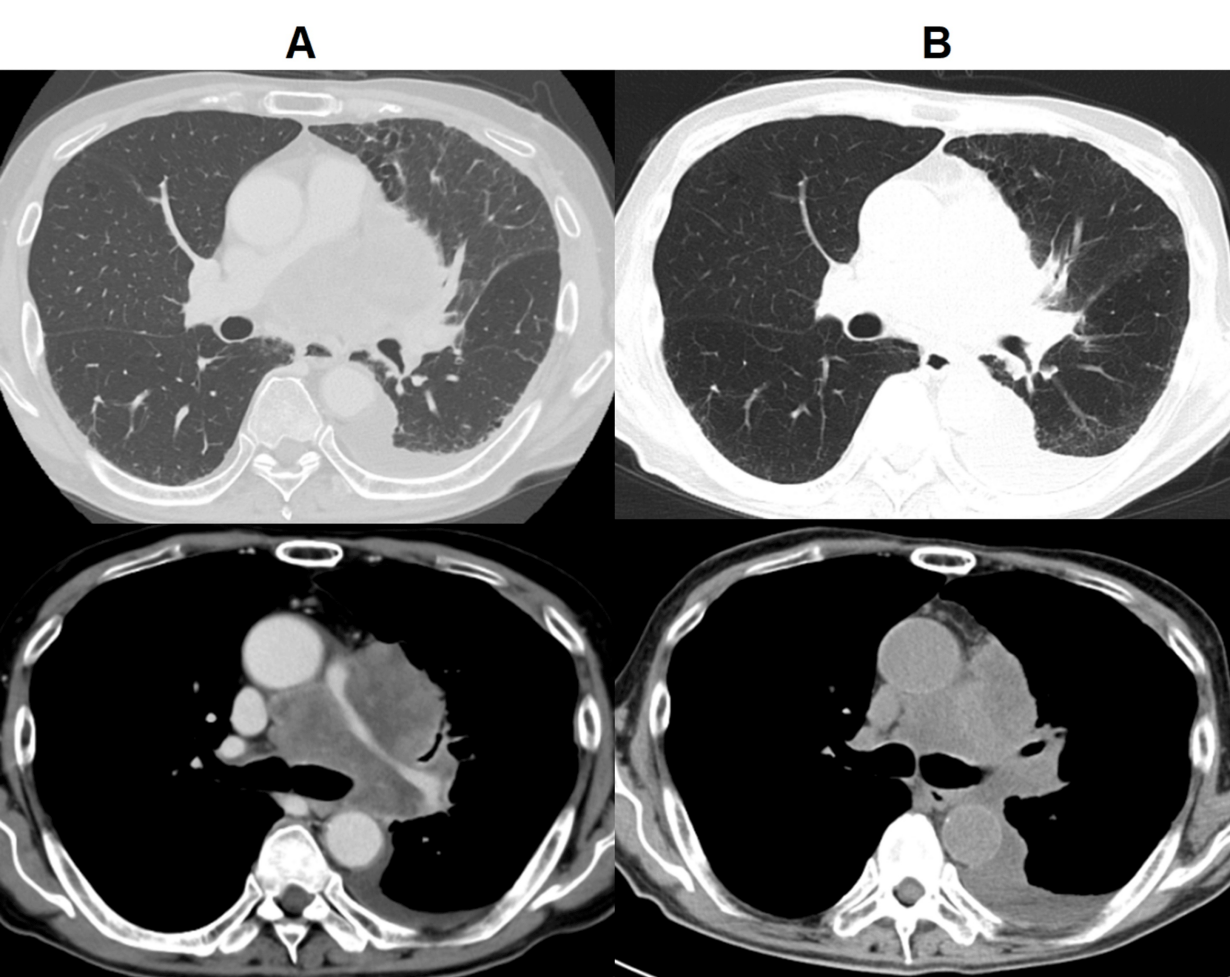
Treatment response Computed tomography (CT) scans (A) with contrast before treatment and (B) without contrast on day 29 of paclitaxel therapy, with partial reduction in the size of the mass.

Following diagnosis, the patient was hospitalized for obstructive pneumonia caused by tumor progression. After his condition improved using antibiotic treatment, weekly paclitaxel therapy was initiated. Mild diarrhea was observed as an adverse event. A CT scan on day 29 after initiation of paclitaxel treatment confirmed a partial response, representing a 30% reduction in the major axis of the primary tumor (Figure 5). This follow-up CT was performed without contrast, considering the patient's deteriorating renal function due to anorexia. For the same reason, the sixth weekly administration of paclitaxel was withheld. Three weeks later, the first administration of the second cycle was postponed due to a left-sided pneumothorax, which required a chest drainage procedure. This was presumed to be a complication of tumor necrosis in response to chemotherapy. Ultimately, the patient developed impaired consciousness owing to carcinomatous meningitis and died 66 days from initial presentation.

## Discussion

Primary pulmonary epithelioid angiosarcoma is exceptionally rare, and no standard treatment protocol exists. Ren et al. reported a median overall survival of only two months for patients with multifocal primary pulmonary angiosarcoma [2].

In the present case, a definitive diagnosis was swiftly achieved using EBUS-TBNA, a minimally invasive procedure. This swift and definitive diagnosis allowed prompt therapy initiation. For peripheral lesions without mediastinal lymph node metastasis, more invasive procedures such as percutaneous needle biopsy or surgical biopsy may be necessary. These procedures carry a significant bleeding risk and require careful consideration in their diagnostic approach.

A definitive diagnosis was made based on histopathological and immunohistochemical findings. Histologically, epithelioid angiosarcoma is characterized by an invasive lesion composed of large, oval, or round cells with abundant eosinophilic nucleoli. Immunohistochemically, CD31, CD34, and factor VIII-related antigen are specific endothelial markers. Owing to its epithelioid nature, tumors generally also test positive for cytokeratin and the mesenchymal marker vimentin. These characteristics allow for the differentiation of epithelioid angiosarcoma from other entities such as epithelioid hemangioendothelioma and metastatic carcinoma [5].

Surgical resection is preferred for localized lesions; however, for unresectable cases, radiotherapy or chemotherapy may be selected [6]. In our patient, surgery and radiotherapy were not indicated because of the presence of multiple metastases. The choice of chemotherapy in the present case required careful consideration based on the patient's condition. Combination therapies, such as platinum-based regimens or gemcitabine plus docetaxel, were deemed unsuitable due to the high risk of severe myelosuppression, fatigue, or nephrotoxicity in a patient with poor performance status and significant anorexia. Vascular endothelial growth factor (VEGF) inhibitors, while mechanistically relevant, showed contraindications due to the tumor's proximity to major blood vessels and a history of gastric ulcers, posing a high risk of hemorrhage. While immune checkpoint inhibitors (ICIs) have shown promise in some angiosarcomas, evidence in primary pulmonary angiosarcoma is scarce. Given the rapidly progressive nature of the disease, we prioritized a cytotoxic agent with a higher likelihood of response. Consequently, weekly paclitaxel was selected as the optimal choice, as its side effect profile is relatively manageable and the treatment can be administered in an outpatient setting. This chemotherapy is a standard treatment option for advanced or metastatic angiosarcoma, with its efficacy being established in a clinical study [7]. This clinical effectiveness is attributed to a dual mechanism of action, including both cytotoxic and anti-angiogenic effects. Although the patient achieved a partial response, his survival was ultimately limited to 66 days from initial presentation. It is important to interpret this outcome in the context of the patient's extremely poor prognosis at baseline, including a poor performance status and a highly aggressive tumor subtype. Therefore, the primary benefit in this case was not a dramatic extension of survival but rather the achievement of an objective tumor response and likely palliation of symptoms, which are critical goals in such a setting. This position weekly paclitaxel as a viable and rational option for frail patients for whom more aggressive therapies show contraindications. This outcome may be partly explained by paclitaxel's limited penetration into the cerebrospinal fluid [8], which likely resulted in a failure to prevent the development of carcinomatous meningitis. Recent reports have indicated that ICIs are effective at treating angiosarcomas at other sites, such as the skin [9] and nasal cavity [10]. The potential role of immunotherapy in epithelioid angiosarcoma, therefore, warrants further investigation.

## Conclusions

Primary pulmonary epithelioid angiosarcoma is a challenging malignancy with a poor prognosis. This case demonstrates that paclitaxel monotherapy can achieve a significant objective radiological response. In patients with poor performance status for whom aggressive combination therapies are contraindicated, achieving tumor shrinkage itself is a clinically meaningful outcome, as it may lead to symptom palliation and maintenance of quality of life. Therefore, we propose paclitaxel as a rational, relatively well-tolerated therapeutic option in this specific clinical scenario, serving as a valuable benchmark for individualized treatment decisions. Further exploration of potential therapies, including ICIs, is necessary to advance personalized treatment strategies for this aggressive disease.
